# Active Thermoplastic Starch Film with Watermelon Rind Extract for Future Biodegradable Food Packaging

**DOI:** 10.3390/polym14163232

**Published:** 2022-08-09

**Authors:** Tatsaporn Todhanakasem, Chayanit Jaiprayat, Thunchanok Sroysuwan, Supakanya Suksermsakul, Rachit Suwapanich, Kamolnate Kitsawad Maleenont, Piyawit Koombhongse, Briana M. Young

**Affiliations:** 1 School of Food Industry, King Mongkut’s Institute of Technology Ladkrabang, Bangkok 10520, Thailand; 2 Wesense Solution Co., Ltd., 17 Moo 2 Soi Ngamwongwan 7, Bangkrasaw, Nonthaburi 11000, Thailand; 3 National Metal and Materials Technology Center (MTEC), Pathumthani 12120, Thailand; 4 Department of Medical Microbiology and Immunology, School of Medicine, University of California at Davis, One Shields Ave, Davis, CA 95616, USA

**Keywords:** thermoplastic starch, watermelon rind extract, biodegradable, mechanical strength, active bio-packaging

## Abstract

Petrochemical plastic wastes generate serious environmental problems because they are resistant to natural decomposition. The aim of this study was to develop a biodegradable active thermoplastic film composed of polyvinyl alcohol (PVA), corn starch (ST), glycerol, and the active compounds from watermelon rind extract (WMRE), or PVA/ST/WMRE, using the casting technique. The film was examined for its mechanical, antioxidant, and functional properties against selected foodborne pathogens. The results showed that the addition of 10% *v*/*v* of watermelon rind extract to the film formulation significantly increased the tensile strength from 19.44 ± 0.84 MPa to 33.67 ± 4.38 MPa and slightly increased the percent elongation at break (% EAB) from 35.04 ± 0.96% to 35.16 ± 1.08%. The antioxidant property of PVA/ST/WMRE film was analyzed based on the DPPH scavenging activity assay, which significantly increased from 29.21 ± 0.24% to 63.37 ± 4.27%. The minimum inhibitory concentration (MIC) of watermelon rind extract was analyzed for the growth inhibition of *Bacillus cereus* ATCC 11778, *Escherichia coli* ATCC 8739, and *Salmonella enterica* subsp. *enterica* serovar Typhimurium ATCC 13311, with 10% (*v*/*v*) found as an optimal concentration against *B. cereus*. Wrapping fresh-cut purple cabbage with PVA/ST/WMRE film significantly reduced the microbial load after 3 days of storage, in comparison to commercial packaging (PET) and thermoplastic control film. Consumer testing of the packaging film indicated that user acceptance of the product was favorable. Therefore, we suggest that this newly developed film can be used as a biodegradable food packaging item that will lead to enhanced food safety, food quality, prolonged shelf life, and consumer acceptance for further food applications.

## 1. Introduction

The global consumption of plastics is more than 200 million tons per year, with an annual growth of approximately 9%. Food packaging plastics account for over half of the total global plastic usage. Most food packaging plastic is single use and is used on-the-go, with the bulk of these non-recyclable or non-biodegradable food package wastes creating a drastic environmental problem on land and in water. As such, there is a growing environmental awareness of the need for food packaging film to meet eco-friendly benchmarks. In addition to better ecological degradability, the films must also meet the requirement to reduce food loss and maintain the quality of food products. Such packaging technology is called active food packaging and can be developed from biopolymeric materials infused with natural active ingredients to function as oxygen scavengers, antioxidants, antimicrobials, ethylene scavengers, and moisture absorbers [1,2,3].

Watermelon (*Citrullus lanatus*) is a summer fruit of the family Cucurbitaceae. It is widely consumed as a refreshing fruit and is grown all over the world. The rind is mostly utilized as an animal feed supplement or fertilizer, and more than 90% of watermelon rind is dumped in the environment, which eventually creates an environmental problem [4]. Watermelon rind comprises 35% of the total watermelon weight and has been used for the production of pectin, pickles and other food products, and as a functional compound to create novel and value added products [5]. It is rich in moisture, protein, fat, minerals, carbohydrates, vitamins, fiber, and potassium [6]. It has a high antioxidant property due to the presence of bioactive compounds, such as L-citrulline, and phenolic compounds, especially benzoic acid, 4 hydroxy, vanillin, saponins, phenols, and alkaloids, which are key active compounds. After ethanol extraction, it exhibits high antioxidant activity by inhibiting potential free radical damage [7]. Watermelon rind extract is also a rich source of antimicrobial compounds, where the antimicrobial activities were mostly correlated with the total phenolic content. The antimicrobial activity was active against various Gram-positive and Gram-negative bacteria, including *Staphylococcus albus, Staphylococcus aureus*, *Escherichia coli*, *Bacillus subtilis*, *Listeria innocua*, and *Klebsiella oxytoca* [8]. It has been reported that the incorporation of watermelon rind extract in pork patties could enhance the shelf life of the product by protecting against various microbial effects after refrigeration [7]. The active compounds that exhibit antioxidant activity from watermelon rind have been widely studied and incorporated into cosmetics, food, and pharmaceutical products [9]. Previous work also demonstrated that biodegradable watermelon rind pectin (WRP) films containing kiwifruit peel extract (KPE) showed excellent tensile strength, Young’s modulus, and retardation of the lipid oxidation of food, when compared to other pectin-based films [10].

Polyvinyl alcohol (PVA) is a synthetic polymer obtained by the hydrolysis of polyvinyl acetate. It has a high capability for forming films, as well as exhibiting high transparency, high sealing ability, high mechanical properties, high thermal stability, and is also high in oxygen and odor barrier properties. The material is biodegradable in composting environments when supplemented with appropriate enzymes and microorganisms [11]. However, PVA film is very fragile and a plasticizer is needed to increase stability. Starch is commonly added to PVA film in order to enhance the water molecular resistance and mechanical properties. Some of these starch additives include tamarind nut powder, coconut shell powder, spent coffee bean powders, and tapioca starch, all of which maintain the biodegradable activity and lower the production costs [12]. Starch-PVA based composite films infused with zinc oxide nanoparticles have also been developed for food packaging applications using the casting technique. The zinc oxide films exhibit enhanced water barrier, mechanical, and antimicrobial properties and have potential usage for food applications [13]. There is also a precedence for including fruit extracts in films. For example, pineapple peel extract, which exhibits antioxidant properties, has been incorporated into PVA-corn starch films for active food packaging [14]. Recently, a push has been made to adapt thermoplastic starch (TPS) to replace synthetic polymers in the packaging field due to its manufacture from raw starch material that is biodegradable, non-toxic, inexpensive, abundantly available, and an alternative solution to meet industrial-scale demands [15,16]. In order to obtain a TPS film, starch granules must be gelatinized with a plasticizer in an excess of water at temperatures lower than 100 °C. In this way, hydrogen bonds are formed between the plasticizer and the starch in an irreversible gelatinization process [17]. The most common plasticizers used include monosaccharides, disaccharides, oligosaccharides, glycerol, xylitol, and sorbitol, all of which improve the tensile strength, stiffness, and water vapor and oxygen barrier properties of the material, while still being safe for use with food products. The chemical structure of the plasticizer and its proportion in the film have a significant effect on the mechanical and barrier properties of TPS films [15]. TPS films are odorless, colorless, transparent, and of very low oxygen permeability, which make them well suited for food products [18]. Previously, antimicrobial biodegradable films based on thermoplastic corn starch and chitosan oligomers (CO) were developed as a packaging prototype for perishable food products and exhibited high potential for inhibiting microbial growth and subsequent food spoilage [19].

The main objective of this work was to create an active PVA-corn starch based TPS film for food packaging purposes using the antimicrobial and antioxidant properties of watermelon rind extract combined with glycerol as a plasticizer. The TPS film was prepared using the casting technique. The use of active films maintains the quality and the freshness of packaged foods during distribution and storage periods, while being a safer alternative for the environment. The active film that we developed was analyzed for its mechanical, antioxidant, and biodegradable capability, along with its antimicrobial properties. The antimicrobial property of the watermelon rind extract was tested against *B. cereus* ATCC 11778, *E. coli* ATCC 8739, and *S.* Typhimurium ATCC 13311 to identify the minimal inhibitory concentration (MIC) prior to incorporation in the active TPS film. The active film was further analyzed for the ability to maintain the quality of fresh cut cabbage based on the microbial load after storage. Finally, we performed consumer testing to determine consumer acceptance of the newly developed film as compared to commercial food packaging.

## 2. Material and Methods

### 2.1. Preparation of the Watermelon Rind Extraction

Watermelon rinds were cut into small pieces and dried in a tray dryer at 50 °C for 24 h. The dried rind was powdered using a blender and further ground into fine particles of 80 mesh powder using a pin mill. The sample was packed in a sealed container and stored in a dry place until used.

The extraction of watermelon rind was performed using an incubator shaker (JSR-JSSI-100T, Gongju-City, Korea) by adding 20 g of watermelon rind powder to a 500 mL Erlenmeyer flask containing 200 mL of 95% ethanol at ambient temperature (35 ± 2 °C) and then agitated at 200 rpm for 24 h. Ethanol was later evaporated in a water bath at 45 °C overnight, and the extract remained in the flask. The extract compound was filtered using Whatman’s filter paper no.1 and stored in amber colored bottles at 4 ± 1 °C for further applications (Pavan Kumar et al., 2018).

### 2.2. Film Preparation

The film was prepared by dissolving 5 g of polyvinyl alcohol (PVA) (Sigma-Aldrich, St. Louis, MI, USA) and 1 g of corn starch into 100 mL of deionized water and homogenized for approximately 4 h at 70 °C using a hotplate stirrer. Then, 25% (*v*/*v*) glycerol was added into the mixture as a plasticizer. Subsequently, 10% (*v*/*v*) of watermelon rind extract was added. The mixture was then dispensed onto acrylic sheets and further dried at 45 ± 2 °C for 8 h. The film was gently peeled off of the acrylic sheet once it dried. PVA film with corn starch and plasticizer was labeled as the control film (PVA/ST), while the film containing the additional watermelon rind extract was labeled as PVA/ST/WMRE. All films were stored in a controlled environment chamber at 24 °C and 50% relative humidity (RH) before further characterization (Pardeep Kumar et al., 2021).

### 2.3. Characterization of Film Physical Properties

The thickness of the film was evaluated using a micrometer (Mitutoyo, Kanagawa, Japan) with measurements taken at 5 arbitrary locations, and the mean value of the films was calculated [12]. Water vapor permeability (WVP) was measured using the applied desiccant method, or cup method, to analyze the barrier properties described by Jahit, et al., 2016 [20]. To this end, the film was cut into a circle with a diameter of 9 cm and placed on an aluminum pan of 5 cm diameter containing 10 g of silica gel (0% RH). Each pan was then weighed and placed in a desiccator containing water at room temperature (35 ± 2 °C). The pan was then weighed every hour for 8 h or until the weight stabilized (±0.0001 g). WVP was calculated using the following equation:

WVTR (g/m^2^·h) = G/T/A = Slope/A (1)

WVP (gm/m^2^·h·mmhg) = WVTR × I /× (P1 − P2) (2)

where G/T represents weight change rate per time obtained from the slope of the graph between time and weight change (g/h).

The weight gained by the film equals the initial weight of the film—the weight after applying the desiccator (g);

I is the average of initial film thickness (mm);

A is the permeation area of the film (m^2^);

(P1 − P2) is the difference in partial pressure of the atmosphere with silica desiccant and pure water. 

Calculated from the equation (P1 − P2) = ∆RH/100, where ∆ RH is the difference between the indoor humidity (%RH = 0) and the initial humidity outside the crucible (%RH = 90), therefore ∆RH = 90.

Tensile strength (TS) and percent elongation at break (%EAB) values of the film were examined by reference to the ASTM D882-10 using the texture analyzer model TA.XT. plus C (Stable Micro Systems, USA) at 10 mm/s [21,22]. The film was cut into 2 cm × 8 cm pieces, and the thickness of each film was measured at 5 different positions to identify the average. The TS of the film and the %EAB were measured as the film’s maximum elongation by a Texture Analyzer [23]. TS was calculated as follows:

Tensile strength (TS) = F_max_/A (3)

where F_max_ is the max load (N) needed to pull the sample apart, and A is the cross-sectional area (m^2^) at the beginning of the test sample.

% EAB was calculated as follows:

%EAB = (l_max_/l0) × 100 (4)

where l_max_ is the film elongation (mm), and I_0_ is the initial grip length (mm). Each experiment was performed on 5 replicated specimens.

### 2.4. Antioxidant Properties of the Film

The DPPH radical scavenging activity was used to analyze the antioxidant property of the film. A total of 20 mg each of the PVA/ST and PVA/ST/WMRE films were cut and immersed in 4 mL of methanol-2,2-diphenyl-1-picrylhydrazyl (DPPH) solution (concentration of 0.1 mM) (Sigma-Aldrich, USA). For watermelon rind extract, 0.1 mL of the extract was mixed with 4 mL of DPPH solution. The samples were incubated at room temperature for 24 h. The absorbance at OD_517_ of all samples were monitored (Jridi et al., 2014). The experiment was performed in triplicate. The percentage of DPPH scavenging activity of the films and the extract was evaluated using the equation:

DPPH scavenging activity (%) = (A_DPPH_ − A_Sample_/A_DPPH_) × 100 (5)

A_DPPH_ is the absorbance at OD_517_ of DPPH. A_Sample_ is the absorbance at OD_517_ of the identified sample.

### 2.5. Antimicrobial Properties of the Extract

Watermelon rind extracts were tested for inhibition of the growth of bacterial strains *B. cereus* ATCC 11778, *E. coli* ATCC 8739, and *S. enterica* subsp. enterica serovar *Typhimurium* ATCC 1331 using 96-well plates to identify the minimum inhibitory concentration (MIC). The concentrated watermelon rind extract was serially diluted from 10% to 0.3125% (*v*/*v*) and mixed with 10% *v*/*v* inoculum of the above foodborne pathogenic strains in nutrient broth (NB). Sterile distilled water was used as a negative control, and ampicillin (150 µg/mL) was used as a positive control. The total volume in the wells was 100 µL. The plate was incubated at 37 °C for 24 h, and the bacterial growth was monitored at OD_600_ using a microplate reader spectrophotometer.

### 2.6. Antimicrobial Properties of Film

PVA/ST/WMRE (containing 10% *v*/*v* watermelon rind extract) film was tested for antimicrobial properties by monitoring the microbial count of fresh-cut purple cabbage during storage. The purple cabbage was cleaned by soaking it in 0.5% (*v*/*v*) vinegar for 10 min and then rinsed with tap water. The cabbage was dried and cut into small pieces. The fresh-cut cabbage was placed into 3 different test containers; polyethylene terephthalate (PET) with a lid (commercial package), PET wrapped with PVA/ST, and PET wrapped with PVA/ST/WMRE. The packed containers were refrigerated at 4 °C for 3 consecutive days. The cabbage from each storage condition was sampled daily to analyze the microbial count by serial dilution and spreading on nutrient agar (NA).

### 2.7. Sensory Quality Assessment of Purple Cabbage for Consumer Perception

The sensory evaluation of purple cabbage at 3 days post-storage was evaluated. The purple cabbage was cut into small pieces and placed into 3 different test containers; polyethylene terephthalate (PET) with a lid (commercial package), PET wrapped with PVA/ST, and the PET wrapped with PVA/ST/WMRE to analyze the color, freshness, and overall visual acceptability of the fresh cut cabbage after 3 days refrigeration at 4 °C and compared with a blind control (fresh-cut purple cabbage). The quality assessment of the storage was tested by 8 semi-trained panelists using a difference from control test and testing acceptance was evaluated by 50 general consumers using a 9-point hedonic scale (1 = extremely dislike, 5 = neither like nor dislike, 9 = extremely like) [24]. The sensory evaluation was performed by 8 semi-trained representative panelists by discussing the quality changes using a scoring system from 1 to 5 with the score of 3 as the cut off for marketing (Table 1), which was adapted from [24].

### 2.8. Biodegradable Analysis

The biodegradation behavior of PVA/ST and PVA/ST/WMRE film (Length 9.5 cm) was studied by ascertaining the dimension during a soil burial test for 35 days under 5 cm depth.

### 2.9. Statistical Analysis

All experiments were performed in triplicate, and the data was statistically analyzed by ANOVA with Duncan’s multiple range, with the exception of the sensory quality assessment, where the statistical analysis was performed by ANOVA with Dunnett’s test for the difference from control test and Duncan’s multiple range for the acceptance test.

## 3. Result and Discussion

**Figure 1:**
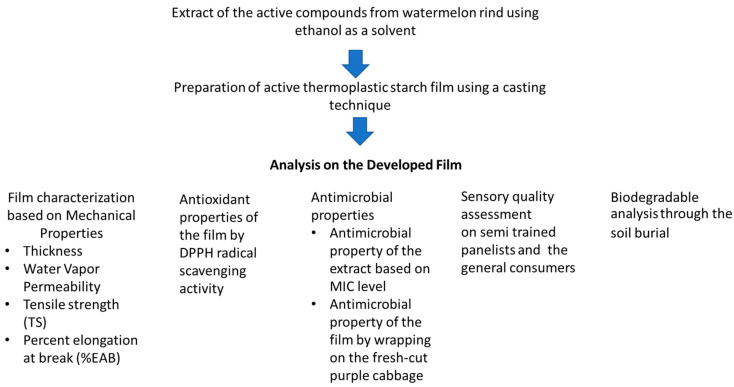
Flowchart of experimental design.

**Figure 2:**
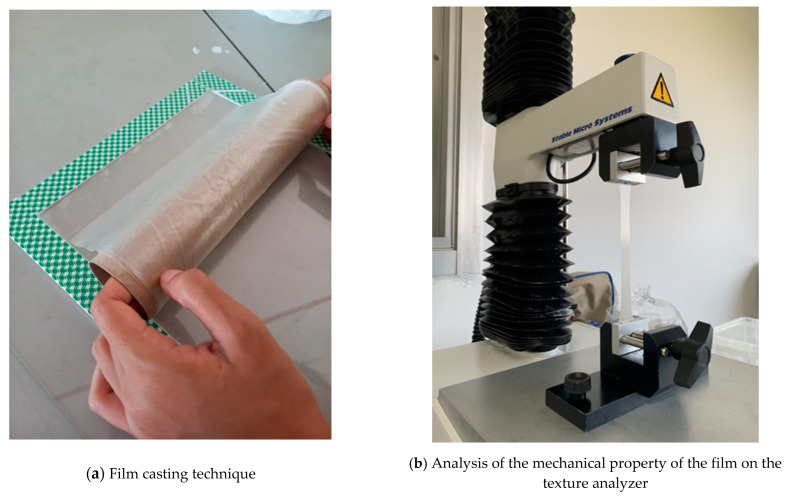
Casting the PVA/ST/WMRE film (**a**) and analysis of the mechanical property of the film on the texture analyzer (**b**).

The development of a novel packaging system that maintains the quality of food with shelf-life extension, as well as reducing environmental effects, has become a big challenge for scientists and the food processing industries. In this study, we developed an active TPS film containing PVA, corn starch, and excessive glycerol as a plasticizer using the film casting technique, with the addition of natural active compounds extracted from watermelon rind (overall protocol shown in Figure 1 and film development in Figure 2). The newly developed active film aims in extending the shelf life and preserves the quality of food products, while also being biodegradable. The active film was characterized for its mechanical, biochemical, and microbiological safety, its sensory attributes, and its capacity to biodegrade.

Watermelon rind extract (10% *v/v*) was incorporated into PVA corn starch, with 25% glycerol added as a plasticizer, in order to create an active food packaging film. The thickness, WVP, TS, % EAB, and antioxidant capacity of the film were investigated. Corn starch and PVA are comprised of many hydroxy functional groups, which are likely to produce intermolecular and intramolecular hydrogen bonding, which greatly influence water molecule resistance properties and mechanical properties. The mechanical strength of films can be firstly controlled by thickness, and as such, it is important to measure the thickness of the films. The PVA/ST/WMRE biopolymer films were found to be of uniform thickness and, after drying, could be smoothly peeled from the acrylic sheets. The thickness of PVA/ST/WMRE was not significantly different from the control (PVA/ST); hence, WMRE had no effect on the film thickness (Table 2). Similar results revealed that the addition of pitanga leaf extract and pineapple peel extract had no significant effect on the thickness of cassava starch-chitosan film and poly (vinyl alcohol)-corn starch film [14,25]. In contrast, film thickness could be increased by the addition of some other extracts, such as beetroot, blueberry, and elderberry extracts [26].

**Figure 3:**
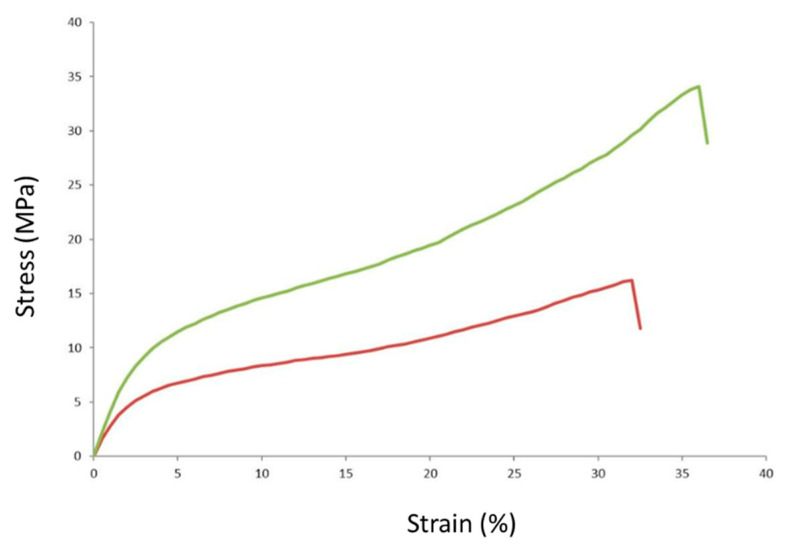
Stress–strain curves of PVA/ST/WMRE (top) and control PVA/ST (bottom) films.

Elasticity, strength, flexibility, and water vapor permeability are also very crucial in the development of food packaging material to maintain the food product’s structural quality and shelf life [27]. The tensile strength of a film can be defined as the maximum force per unit area that a film can endure before breaking, while the flexibility and extensibility of film can be represented by % EAB. The stress–strain curves of selected PVA/ST/WMRE and control PVA/ST are shown in Figure 3. Both films show a plastic deformation area, which is defined by the linear relationship between stress and strain before it undergoes plastic deformation. The TS of PVA/ST/WMRE film is higher than that of PVA/ST, while the % EAB is not much different between the two (Table 2). The TS of PVA/ST/WMRE was significantly higher than PVA/ST (*p* < 0.05), which was about 1.7 times higher than the control, while the % EAB of the two samples was similar. The TS was increased when watermelon peel extract was added to the film, and we hypothesize that this was a result of the alteration of the biopolymer arrangement. It has been previously reported that the crosslinking generated between the amino groups of proteins and phenolic groups available in WMRE results in the increased rigidity of the films, subsequently increasing the tensile strength. This was also seen when mango kernel extract was added to starch films [28]. However, WMRE maintained the % EAB similar to the control. In our study, both PVA/ST and PVA/STWMRE films were developed as TPS that requires the addition of high plasticizer content (glycerol 15–32%), and the film was produced under the appropriate elevated temperature and stirring conditions [29]. The plasticizers commonly used today are hydrophilic low-molecular-weight polyols, including glycerol [30]. The nature of the plasticizer system greatly affects the transformation of granular starch into a homogeneous thermoplastic phase. TPS, in the presence of an appropriate plasticizer, a tiny amount of water, and the application of sufficient thermomechanical energy, causes hydrogen bonds between the starch molecules to be destroyed, while new hydrogen bonds between the plasticizer and starch molecules are synchronously formed and homogenized, thereby improving the mechanical property of the material [31]. Our PVA/ST and PVA/STWMRE films were produced using this concept, with an excess of added glycerol (25%) to increase the tensile strength of the material (up to 2.4 times higher) and the addition of WMRE to further increase the TS (4.2 times higher) when compared to other reported thermoplastic based starches [17]. The % EAB of PVA/ST/WMRE was not significantly different when compared to PVA/ST. However, the addition of excessive plasticizer to PVA/ST and PVA/STWMRE films did result in higher % EAB. Previous work has shown that the addition of 25% glycerol results in the drastically increased flexibility of starch films, while the strength is reduced [32].

Food products are more susceptible to spoilage when they are exposed to high moisture. Therefore, it is necessary for food packaging to have a good barrier towards WVP. As shown in Table 2, the incorporation of WMRE did not affect the WVP of the film, as compared to the control film, where the WVP of both materials was around 6.5 g.m/m^2^.h.mmHg. Hence, the incorporation of WMRE did not appreciably change the hydrophobic nature of the film [33]. Instead, the addition of a high percentage of glycerol seemed to increase the WVP of the films. Glycerol is a relatively small hydrophilic molecule that can be inserted between adjacent polymeric chains, decreasing intermolecular attractions and increasing molecular mobility, which facilitates the migration of water vapor molecules [34].

**Figure 4:**
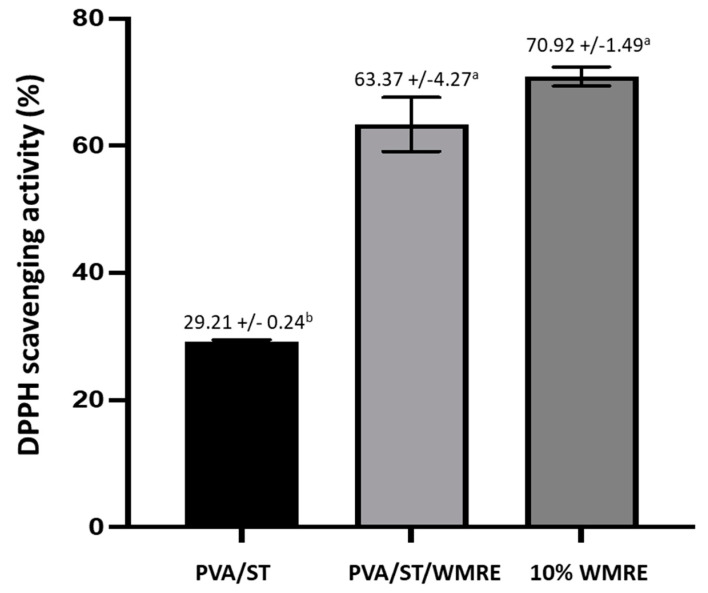
DPPH scavenging activity of PVA/ST, PVA/ST/WRE, and 10% *v*/*v* watermelon rind extract. The different letters represent a significant difference of *p* < 0.05. The error bar indicates the standard deviation (n = 3).

Recently, various natural and synthetic antioxidant agents have been added directly to processed food products during manufacturing to delay the auto-oxidation of lipids in food. However, active antioxidant packaging systems are an attractive alternative to adding compounds directly into food [35]. Watermelon rind extract can function as an active antioxidant in films and therefore, could replace the usage of artificial antioxidants that are more strictly regulated, as they cause a greater health risk and are gradually becoming unacceptable to customers because of their possible toxicity [36,37]. Of the total watermelon by-products, watermelon rind contains the highest total phenolic content at 0.026 ± 0.003 mg GAE/g [8]. The functional compounds present in the WMR are composed of alkaloids, phytates, tannins, oxalates, phenols, flavonoids, and saponin [38]. A comparison of the DPPH scavenging activity of PVA/ST, PVA/ST/WRE, and watermelon rind extract (10% *v*/*v*) (Figure 4) indicates that watermelon rind extract (10% *v*/*v*) itself has a 70.92 ± 1.49% DPPH scavenging activity, but when added to the PVA/ST film and developed as PAV/ST/WRE, there is a slight reduction in the percent of DPPH scavenging activity; however, the differences were not significant. In contrast, the DPPH scavenging activity of PVA/ST/WRE was significantly different from that of the PVA/ST. Therefore, the incorporation of WMRE into PVA/ST film could improve the antioxidant activities of the film. In a previous study, watermelon rind exhibited a 34.48% DPPH scavenging activity [8]. This activity could be explained by the presence of phenolic compounds in watermelon rind extract associated with metal ion-chelation and radical scavenging activities, which lead to their antioxidant abilities when mixed with the film [39].

**Figure 5:**
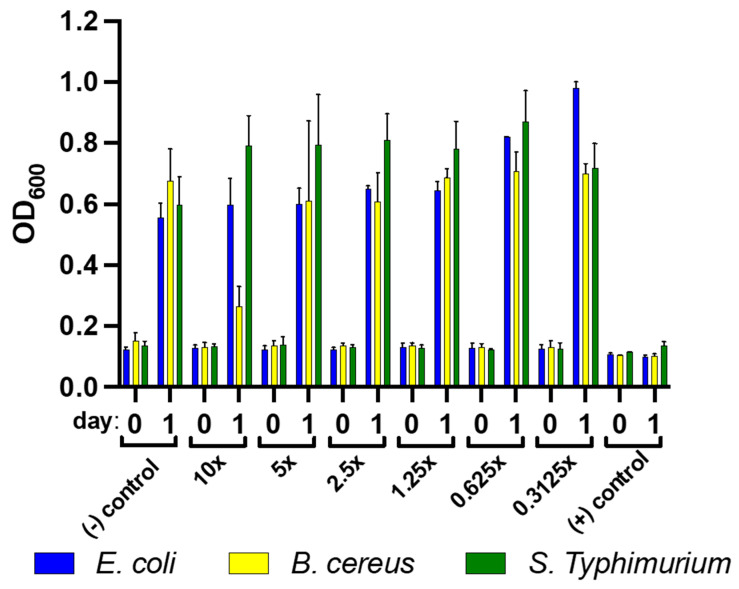
Antimicrobial properties of watermelon rind extract in 96-well plates at concentrations from 10–0.3125% against *B. cereus*, *E. coli*, and *S. enterica* subsp. enterica serovar *Typhimurium* when incubated at 37 °C for 24 h. The growth was monitored at OD6_00_ using a microplate reader spectrophotometer. The error bars indicate the standard deviation (n = 3).

**Figure 6:**
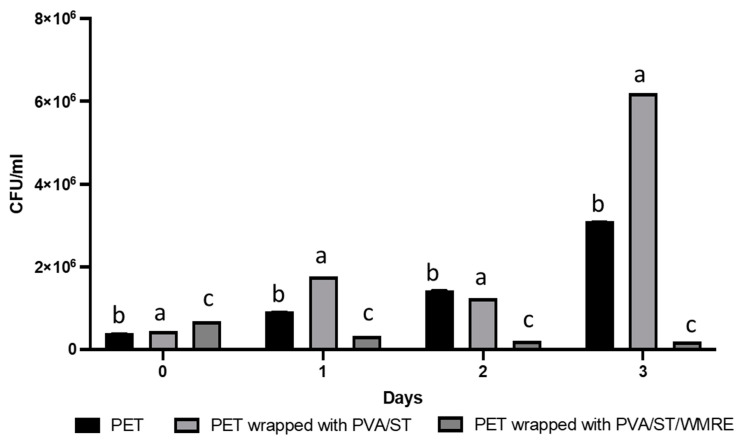
Microbial count, as colony forming units (CFU/mL), of fresh-cut purple cabbage under three different conditions of storage: commercial package with a lid (PET), PET wrapped with PVA/ST, and PET wrapped with PVA/ST/WMRE, while refrigerated at 4 °C. Analysis was performed on 3 consecutive days. The different letters represent a significant difference of *p* < 0.05. The error bars indicate the standard deviation (n = 3).

The antimicrobial properties of the experimental films were also investigated. The MIC of WMRE (10% *v*/*v*) was analyzed in 96-well plates at different serial dilutions from 10%–0.3125% *v*/*v* against *B. cereus*, *E. coli,* and *S. enterica* subsp. enterica serovar *Typhimurium* (Figure 5). The growth inhibition on the tested pathogens was monitored by optical density (OD_600_). The growth inhibition was found to affect *B. cereus* only on day 1 and only at 10% concentration, while there was no effect on *E. coli* and *S. enterica* subsp. Enterica serovar *Typhimurium*. Watermelon rind has been reported to inhibit the growth of *B. cereus* and *E. coli* with an MIC of 8.0 mg/mL and 128 mg/mL, respectively, when extracted with methanol [8]. The microbial count as colony forming units (CFU/mL) of fresh-cut purple cabbage was analyzed when stored at 4 °C on 3 consecutive days under 3 different storage conditions: PET with a lid or commercial package, PET wrapped with PVA/ST, and PET wrapped with PVA/ST/WMRE (Figure 6). The microbial count on the fresh-cut cabbage gradually increased when stored in PET with the lid and PET wrapped with PVA/ST. In contrast, the microbial count on fresh-cut purple cabbage was slightly reduced throughout day 3, when wrapped with PVA/ST/WMRE. At the starting time, the microbial counts were similar among the 3 different conditions; however, after 3 days of storage, PVA/ST/WMRE wrap was more effective in inhibiting the microbial growth on the fresh-cut purple cabbage. Therefore, PVA/ST/WMRE could effectively delay the rapid increase in total viable microbes on fresh-cut vegetables and may help to prolong the shelf life of the product, especially in terms of microbial food spoilage.

We evaluated consumer acceptance of the food quality when the film was applied to fresh-cut purple cabbage by comparing the quality with commercial food packaging. The quality assessment based on the color and freshness of the fresh-cut purple cabbage when stored at 4 °C for 3 days in PET with a lid (commercial packaging), PET wrapped with PVA/ST, PET wrapped with PVA/ST/WMRE, and the blind control using the difference from control test are shown in Table 3 and Table 4. A semi-trained panel evaluated the samples in order to assess the degree of changes in fresh-cut cabbage upon storage, according to human perception. While instrumental measures may be crucial for the detection of quality loss during the shelf life of products, they may not be relevant for consumer acceptability [40]. The scores were based on a scoring system from 1–5, with the score of 3 as the cut off for the turnover point of shelf life. The fresh-cut purple cabbages were not significantly different from the control in both color and freshness qualities (*p* ≥ 0.05). The new wrap using extracts from watermelon did not affect the fresh-cut purple cabbage negatively, and there were no perceivable differences between such wrap and the control. Additionally, consumer acceptance of the fresh-cut cabbage stored at 4 °C for 3 days in PET with a lid, PET wrapped with PVA/ST, and PET wrapped with PVA/ST/WMRE were determined using a 9-point hedonic score. General consumers who purchase fresh-cut produce participated in the test in order to measure the acceptability of the samples. Table 5 illustrates that the mean scores of color of the samples was not significantly different in terms of liking on day 0 and day 1 (*p* ≥ 0.05). Significant differences in mean liking scores between the 3 samples were clearly seen on day 2 (*p* < 0.05), where PET wrapped with PVA/ST/WMRE was significantly liked, followed by PET with a lid, and PET wrapped with PVA/ST. The same pattern was seen on day 3. Yet, it should be noted that all samples exhibited good color quality with regards to consumer acceptance, since the liking scores range from like moderately (score of 7 out of 9) to like very much (score of 8 out of 9). Consumer preference for the freshness of fresh-cut cabbage stored in different types of packaging was not significantly different on day 0 (*p* ≥ 0.05) (Table 6). However, on days 1, 2, and 3, the mean liking score for fresh-cut cabbage wrapped with PVA/ST/WMRE was significantly higher than those stored in PET with a lid and PET wrapped with PVA/ST (*p* < 0.05).

**Figure 7:**
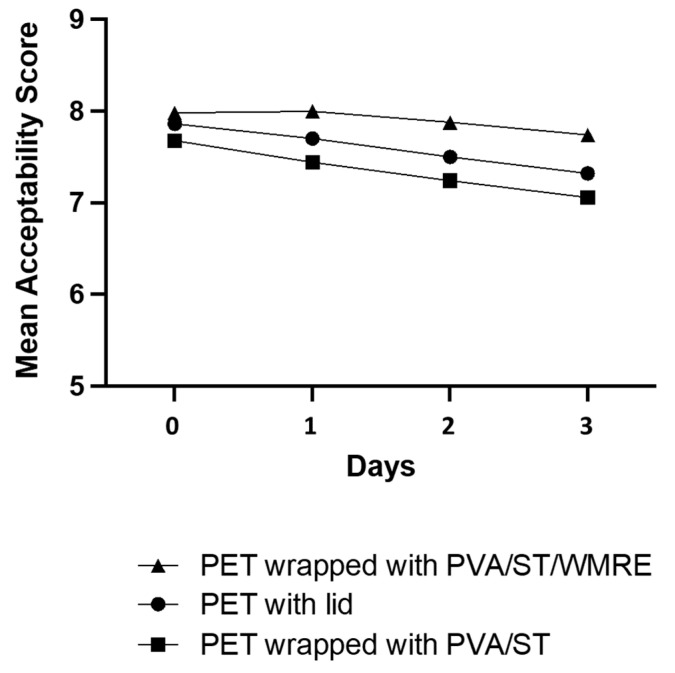
The overall acceptability score of the three different conditions of storage: PET with a lid, PET wrapped with PVA/ST, and PET wrapped with PVA/ST/WMRE while refrigerated at 4 °C, with analysis on 3 consecutive days.

When comparing changes in the mean liking score of color within the duration of 3 days, only the score for the fresh-cut purple cabbage stored in PET wrapped with PVA/ST/WMRE was found to be insignificant (*p* ≥ 0.05). A significant decrease in mean liking score for fresh-cut cabbage stored in PET with a lid was found on day 3, while scores for the cabbage stored in PET wrapped with PVA/ST decreased on day 2 (*p* < 0.05). The preference for fresh-cut purple cabbage stored in PET with a lid and PET wrapped with PVA/ST/WMRE did not change from day 0 to day 3. On the other hand, fresh-cut cabbage stored in PET wrapped with PVA/ST showed a significant decrease in liking on day 1 (*p* < 0.05). The mean liking score for overall visual acceptability of fresh-cut purple cabbage stored in PET wrapped with PVA/ST/WMRE was significantly higher than for that stored in the other two kinds of packing, as evaluated on days 1, 2, and 3 (*p* < 0.05) (Table 7). It was only found on day 0 that the mean liking scores for PET wrapped with PVA/ST/WMRE and PET with a lid were not significantly different (*p* ≥ 0.05). The changes in mean liking scores for the overall visual quality of fresh-cut purple cabbage for the duration of 3 days were in line with the results of freshness for those stored in PET with a lid and PET wrapped with PVA/ST/WMRE and did not exhibit a decrease in preference. The consumer preference for fresh-cut cabbage stored in PET wrapped with PVA/ST started to significantly decrease on day 3 (*p* < 0.05). The mean overall acceptability score for fresh-cut purple cabbage stored in 3 different conditions were in the range of 7–8 out of 9 points, which translated to like moderately to like very much (Figure 7). Such acceptability scores indicated that within the turnover period of fresh-cut produce, the samples stored in all three conditions maintained their acceptability to consumers. Therefore, fresh-cut purple cabbage stored in PET wrapped with PVA/ST/WMRE received the highest acceptability score when compared to other packaging methods, in all storage days. Wraps with watermelon extract did not impose any negative effects on consumer perception. As a matter of fact, the consumers perceived the quality of fresh-cut purple cabbage stored in PET wrapped with PVA/ST/WMRE to be better than those stored conventionally.

**Figure 8:**
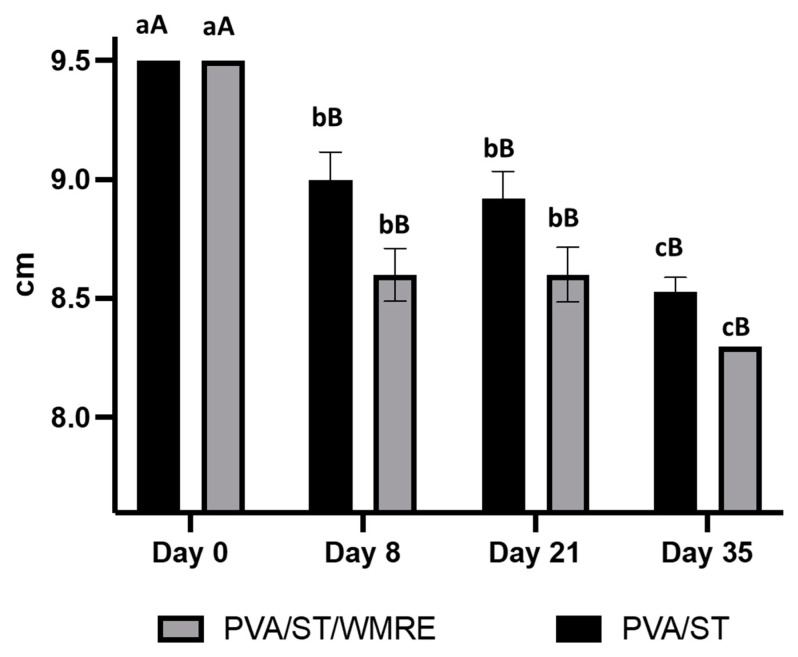
Biodegradation capability based on the length of PVA/ST and PVA/ST/WMRE films when buried in soil at 5 cm depth for 35 days. The different letters represent a significant difference of *p* < 0.05. The error bar indicates the standard deviation (n = 3). Lowercase denotes significant difference within the day (*p* < 0.05). Uppercase denotes significant difference within the type of film (*p* < 0.05).

PVA/ST and PVA/ST/WMRE were also analyzed for biodegradable capability by burying samples in soil (at 5 cm depth) for 35 days and subsequently monitoring the length of the film (Figure 8). The capacity of PVA/ST and PVA/ST/WMRE to biodegrade was analyzed based on the length of the film and was found to be significantly different from day 8 onward. There were no significant differences in the capability of the two films to biodegrade on each specific day. After 35 days of soil burial, a total of 12.6% of PVA/ST/WMRE and 10.21% of PVA/ST were degraded when compared to the original length on day 0. Previous work has illustrated the high potential of PVA/starch based polymers to easily biodegrade when buried in soil. Similar results have also been found with PVA and cassava starch and PVA mixed with other starch types [41,42].

## 4. Conclusions

Active packaging is an excellent solution for a wide range of applications in the food industry. The most important advantages resulting from their use are the extension of food shelf life and the reduction in food loss. In this study, we evaluated the presence of excessive plasticizer (glycerol) in TPS and found it could improve TS, %EAB, and WVP properties of PVA/ST and PVA/ST/WMRE, especially when combined with the addition of 10% WMRE in PVA/ST/WMRE. The incorporation of WMRE in TPS made a significant improvement in the TS of the film (1.7 time higher). Our study demonstrated a significant benefit of PVA/ST/WMRE in terms of super antioxidant capacity (63.37% DPPH scavenging activity) and higher antimicrobial properties against selected foodborne pathogens. These films can be used to mitigate food spoilage and extend the shelf life and quality of fresh-cut vegetables. Additionally, the application of the film had no effect on the sensory attributes of fresh-cut vegetables. The properties of the newly developed film were comparable to commercial food packaging (PET), but with the benefit that this film also had a high biodegradable capability, where 12.6% of the material can be biodegraded within 35 days. This work demonstrated the efficacy of PVA/ST/WMRE as an active TPS film for further application as food packaging, with less environmental impact.

## Figures and Tables

**Table 1:** Quality assessment scoring system for fresh-cut purple cabbage.

Characteristics	Score				
	5	4	3	2	1
Color (cut surface browning)	None	Traces in <5 pieces	Very light in <10 pieces	Light in >10 pieces	Dark in >10 pieces
Freshness	Very fresh	Fresh	Marketable	Edible non marketable	Non edible

**Table 2:** Mechanical properties of PVA/ST and PVA/ST/WMRE films. The different letters represent a significant difference of *p* < 0.05. The error bar indicates the standard deviation (n = 3).

Parameter	PVA/ST	PVA/ST/WMRE
Thickness (mm)	0.0063 ± 0.0021 ^a^	0.0059 ± 0.0019 ^a^
Tensile strength (MPa)	19.44 ± 0.84 ^b^	33.67 ± 4.38 ^a^
Elongation at break (%)	35.04 ± 0.96 ^a^	35.16 ± 1.08 ^a^
Water vapor permeability (WVP) (g.m/m^2^.h.mmHg)	6.55 ± 1.032 ^a^	6.59 ± 0.917 ^a^

Note: Different lowercase superscripts denote significant difference within the column (*p* < 0.05).

**Table 3:** The quality assessment on color of fresh-cut purple cabbage.

Color				
Sample	Day 0 ^ns^	Day 1 ^ns^	Day 2 ^ns^	Day 3 ^ns^
PET with lid	4.9 ± 0.4	4.8 ± 0.5	4.9 ± 0.35	4.9 ± 0.4
PET wrapped with PVA/ST	4.9 ± 0.4	4.9 ± 0.4	4.9 ± 0.35	4.9 ± 0.4
PET wrapped with PVA/ST/WMRE	4.9 ± 0.4	4.9 ± 0.4	4.9 ± 0.35	4.8 ± 0.5
Blind control	4.8 ± 0.5	4.9 ± 0.4	4.8 ± 0.46	4.9 ± 0.4

Note: ^ns^ represents a non-significant difference of *p* ≥ 0.05.

**Table 4:** The quality assessment on freshness of fresh-cut purple cabbage.

Freshness				
Sample	Day 0 ^ns^	Day 1 ^ns^	Day 2 ^ns^	Day 3 ^ns^
PET with lid	4.9 ± 0.4	4.8 ± 0.5	4.9 ± 0.4	4.9 ± 0.4
PET wrapped with PVA/ST	4.7 ± 0.5	4.9 ± 0.4	4.6 ± 0.5	4.9 ± 0.4
PET wrapped with PVA/ST/WMRE	4.9 ± 0.4	4.9 ± 0.4	4.9 ± 0.4	4.8 ± 0.5
Blind control	4.6 ± 0.5	4.8 ± 0.5	4.6 ± 0.5	4.9 ± 0.4

Note: ^ns^ represents a non-significant difference of *p* ≥ 0.05.

**Table 5:** Mean liking score and standard deviation of color of fresh-cut purple cabbage (n = 50).

Color				
Sample	Day 0	Day 1	Day 2	Day 3
PET with lid	7.8 ± 1.1 ^bA^	7.9 ± 0.9 ^aA^	7.64 ± 0.9 ^bA^	7.3 ± 1.2 ^bB^
PET wrapped with PVA/ST	7.6 ± 1.3 ^bAB^	8.0 ± 1.1 ^aA^	7.24 ± 1.18 ^B^	6.9 ± 1.5 ^cC^
PET wrapped with PVA/ST/WMRE^NS^	8.1 ± 1.0 ^a^	8.1 ± 0.8 ^a^	8.00 ± 1.0 ^a^	7.9 ± 1.2 ^a^

Note: Different lowercase superscripts denote significant difference within a column (*p* < 0.05). Different uppercase superscripts denote significant differences within a row (*p* < 0.05).

**Table 6:** Mean liking score and standard deviation of freshness of fresh-cut purple cabbage (n = 50).

Freshness				
Sample	Day 0	Day 1	Day 2	Day 3
PET with lid^NS^	7.8 ± 1.1 ^a^	7.6 ± 1.4 ^b^	7.4 ± 1.1 ^b^	7.36 ± 1.24 ^b^
PET wrapped with PVA/ST	7.8 ± 1.3 ^aA^	7.3 ± 1.3 ^cB^	7.2 ± 1.2 ^bB^	6.86 ± 1.59 ^cB^
PET wrapped with PVA/ST/WMRE^NS^	7.9 ± 1.2 ^a^	8.0 ± 1.2 ^a^	7.8 ± 1.1 ^a^	7.90 ± 1.31 ^a^

Note: Different lowercase superscripts denote significant difference within a column (*p* < 0.05). Different uppercase superscripts denote significant difference within a row (*p* < 0.05).

**Table 7:** Mean liking score and standard deviation of overall visual acceptability of fresh-cut purple cabbage (n = 50).

Overall				
Sample	Day 0	Day 1	Day 2	Day 3
PET with lid^NS^	7.9 ± 0.9 ^ab^	7.7 ± 1.3 ^b^	7.5 ± 1.3 ^b^	7.3 ± 1.4 ^b^
PET wrapped with PVA/ST	7.7 ± 0.9 ^bA^	7.4 ± 1.3 ^cAB^	7.2 ± 1.1 ^cAB^	7.0 ± 1.5 ^bB^
PET wrapped with PVA/ST/WMRE^NS^	8.0 ± 1.1 ^a^	8.0 ± 1.2 ^a^	7.9 ± 1.2 ^a^	7.8 ± 1.4 ^a^

Note: Different lowercase superscripts denote significant difference within a column (*p* < 0.05). Different uppercase superscripts denote significant difference within a row (*p* < 0.05).

## Data Availability

Not applicable.
